# Hormonal determinants of mammographic density and density change

**DOI:** 10.1186/s13058-020-01332-4

**Published:** 2020-08-26

**Authors:** Marike Gabrielson, Shadi Azam, Elina Hardell, Madeleine Holm, Kumari A. Ubhayasekera, Mikael Eriksson, Magnus Bäcklund, Jonas Bergquist, Kamila Czene, Per Hall

**Affiliations:** 1 Department of Medical Epidemiology and Biostatistics, Karolinska Institutet, Nobels väg 12A, SE-171 77 Stockholm, Sweden; 2 Analytical Chemistry and Neurochemistry, Department of Chemistry – BMC, Uppsala University, Uppsala, Sweden; 3 Department of Oncology, South General Hospital, Stockholm, Sweden

**Keywords:** Mammographic density, Mammographic density change, Plasma hormones

## Abstract

**Background:**

Mammographic density (MD) is a strong risk factor for breast cancer. We examined how endogenous plasma hormones are associated with average MD area (cm^2^) and annual MD change (cm^2^/year).

**Methods:**

This study within the prospective KARMA cohort included analyses of plasma hormones of 1040 women. Hormones from the progestogen (*n* = 3), androgen (*n* = 7), oestrogen (*n* = 2) and corticoid (*n* = 5) pathways were analysed by ultra-performance supercritical fluid chromatography-tandem mass spectrometry (UPSFC-MS/MS), as well as peptide hormones and proteins (*n* = 2). MD was measured as a dense area using the STRATUS method (mean over the left and right breasts) and mean annual MD change over time.

**Results:**

Greater baseline mean MD was associated with overall higher concentrations of progesterone (average + 1.29 cm^2^ per doubling of hormone concentration), 17OH-progesterone (+ 1.09 cm^2^), oesterone sulphate (+ 1.42 cm^2^), prolactin (+ 2.11 cm^2^) and SHBG (+ 4.18 cm^2^), and inversely associated with 11-deoxycortisol (− 1.33 cm^2^). The association between MD and progesterone was confined to the premenopausal women only. The overall annual MD change was − 0.8 cm^2^. Hormones from the androgen pathway were statistically significantly associated with MD change. The annual MD change was − 0.96 cm^2^ and − 1.16 cm^2^ lesser, for women in the highest quartile concentrations of testosterone and free testosterone, respectively, compared to those with the lowest concentrations.

**Conclusions:**

Our results suggest that, whereas hormones from the progestogen, oestrogen and corticoid pathways drive baseline MD, MD change over time is mainly driven by androgens. This study emphasises the complexity of risk factors for breast cancer and their mechanisms of action.

## Introduction

Breast cancer is the most commonly diagnosed cancer in women around the world, and mammographic breast density (MD) is one of the strongest risk factors. MD reflects the radiographically dense fibroglandular tissue, which appears bright on the mammogram. Women with high breast density have a 4–6-fold increased breast cancer risk as compared to women with low density [1–4]. Studies analysing the relation between MD and endogenous plasma hormones have shown inconsistent results [5–13]; it remains largely uncertain how progestogens, oestrogens, androgens and corticoids are associated with MD in the normal, non-malignant breast. MD is a highly inheritable trait, but it is also influenced by well-established lifestyle risk factors for breast cancer [14, 15]. Menopausal hormone therapy (MHT) is used to relieve common symptoms of menopause such as hot flushes, sleeping disturbance, depressive mood and muscle and joint pain. Randomised clinical trials have shown that both MHT with oestrogen alone and oestrogen plus progestin increases the MD in postmenopausal women [16–19]. The Women’s Health Initiative (WHI) study found that postmenopausal women who received combined oestrogen plus progestin significantly increased the incidence of breast cancer within a 5-year period compared to the placebo group [20]. In addition, they showed that the frequency of mammograms with suspicious findings in the oestrogen-plus-progestin group was higher than that in the placebo group. High MD may also lead to masking, thus making it harder to detect tumours in the breast.

MD is a dynamic trait; density decreases with age, a natural biological process called involution [21]. We have previously shown that the overall annual MD change is − 1.0 cm^2^ [15]. In contrast to overall MD, natural MD change is not strongly influenced by typical risk factors for breast cancer, except for BMI and physical activity, although results remain inconclusive for postmenopausal women [15, 22–25]. MD can however be decreased. Studies have shown that the use of the selective oestrogen receptor modulator tamoxifen for prevention of breast cancer induces an MD decrease [26–29]. No studies so far have investigated the association between endogenous plasma hormones and natural MD change.

We have previously developed a method for analysing endogenous plasma steroid hormones by ultra-performance supercritical fluid chromatography-tandem mass spectrometry (UPSFC-MS/MS) [30]. The panels were selected to cover hormones from the progestogen, androgen, oestrogen and corticoid pathways. We used the unique prospective Karolinska Mammography Project for Risk Prediction and Breast Cancer (KARMA) cohort to study the association between the plasma hormones on both MD and MD change over time.

## Methods

### Study population

In this nested study within the large KARMA cohort, we included 1040 clinically healthy controls without any prior breast cancer diagnosis or other cancer and who were not using MHT at the time of blood draw. No women with previous gynaecological surgery were included. The samples were previously randomly selected as age-matched controls to breast cancer cases within the KARMA cohort. KARMA is a population-based prospective cohort study initiated in 2011 comprising 70,877 women attending mammography screening or clinical mammography in Sweden [31, 32]. The overarching goal of KARMA is to reduce the incidence and mortality of breast cancer by focusing on individualised prevention and screening.

Women completed a comprehensive KARMA baseline questionnaire and donated non-fasting EDTA plasma samples of peripheral blood at enrolment [31, 32]. All variables included in the analyses were collected using the web-based questionnaire at study entry. Baseline BMI was self-reported.

Each study participant signed a written informed consent form and accepted linkage to national breast cancer registers. The Stockholm ethical review board approved the study (2010/958-31/1).

### Mammographic density measurements

Processed mammograms from mediolateral oblique and craniocaudal views of the left and right breasts were collected from full-field digital mammography system at study enrolment [31, 32]. We used average dense area (cm^2^) (over the left and right breasts) using the STRATUS area-based method. STRATUS is a fully automated tool developed to analyse digital and analogue images using an algorithm that measures density on all types of images regardless of vendor [33]. When studying repeated mammograms from the same individual women, it is important to consider the technical differences between mammogram. In the current study, mammograms from the same women were aligned before density measures were performed. The concept of the alignment method has been described previously [33], as has the calculation of MD change over time [15].

### Laboratory analyses

All blood samples were collected at study entry and handled in accordance with a strict 30-h cold-chain protocol at the Karolinska Institutet high-throughput biobank. Hormones were measured in blinded peripheral blood plasma by the UPSFC-MS/MS system (Waters Corporation, Milford, USA), as described previously [30]. Sample preparation for the analysis of desulfated steroid hormones was carried out through liquid-liquid extraction with tert-butyl methyl ether (MTBE) followed by derivatisation with methoxyamine. Sulphated DHEA (DHEAS) was analysed directly, after extraction with MTBE after protein precipitation. The separation of the desulfated steroids and DHEAS was accomplished using the Acquity UPC^2^ BEH and CSH fluoro-phenyl columns (3.0 mm × 150 mm, 1.7 μm), respectively (Waters, Milford, USA). Desulfated steroid methoxyamine derivatives were separated using 0.1% formic acid in methanol-isopropanol (1:1, v/v) (2 mL/min) as a modifier whereas DHEAS was separated using 10 mM ammonium acetate in methanol with 3% (v/v) water (1.5 mL/min) using the respective columns. The MS detection was performed using electrospray ionisation in the positive ionisation mode ESI+ve for desulfated steroid methoxyamine and positive ionisation mode ESI-ve for DHEAS derivatives, with nitrogen as desolvation gas and argon collision gas. Data acquisition range was set to *m*/*z* 100–600. The quantification was based on a multiple reaction monitoring (MRM) method; collision energy and cone voltage were set as described previously [30], using individual analysis of standard desulfated steroids and DHEAS (100 ng/mL). Quantification of hormones was performed using the suitable deuterated internal standards, and limit of quantification (LOQ) of desulfated steroids (0.05–0.5 ng/mL) and DHEAS (0.01 ng/mL). The coefficient of variation of desulfated steroids and DHEAS assays was < 7.2% and < 3.2%, respectively. Limit of detection (LOD) and LOQ were determined as the lowest concentration which provided a signal-to-noise ratio (S/N) greater than 3 and 10, respectively, by repeated injection (*n* = 6) with a relative standard deviation of replicates below 15%. Values were missing for all hormones and across all batches. Information on LOQ and linear range for all steroinds can be found in [30]. Data was acquired, analysed and processed using the MassLynx TM4.1 software (Waters, Milford, USA).

The peptide hormones SHBG (cat.no. DSHBG0B) and prolactin (cat.no. DPRL00) were measured by using immuno-assay kits from R&D Systems (Minneapolis, USA). Both sandwich-type assays used a pre-coated 96-well plate and a supply of enzyme-labelled secondary antibody, and standards, and were analysed according to the manufacturer’s instructions. The resulting absorbance was read in a BioRad 680 Microplate Reader (BioRad Laboratories, Hercules, CA) at 450 nm with 595 nm as background. The goodness of fit was verified by the *r*^2^ values. The LOQ was 2.0 nM for SHBG and 0.6 ng/mL for prolactin.

### Statistical analyses

Multivariable adjusted linear regression models were used to estimate the association of endogenous hormones with baseline mean MD and 95% confidence interval (CI), as well as annual MD change (95% CI). Annual MD change over the follow-up period was estimated for each woman as a slope using a linear regression on age at each MD measurement. We calculated the geometric mean of baseline MD or MD change within tertile distributions for each hormone, and the difference between each category and the reference by multivariable-adjusted analyses of variance. *P* for trend was calculated by linear regression with baseline MD or MD change as a dependent continuous variable across tertiles of hormones. All models were adjusted for age and BMI at baseline, time of day of blood draw and plasma sample plate number to account for missingness by technical error. All models for MD change were additionally adjusted for physical activity (MET-h/d) at baseline. Hormones were natural log-transformed. Linear regression models with continuous variables to estimate the association of endogenous hormones with baseline mean MD and annual MD change were also stratified by menopausal status defined at baseline. All *P* values were two-sided and considered statistically significant if < 0.05. Analyses were conducted using SPSS (version 26; IBM Corporation).

## Results

### Baseline characteristics

Baseline characteristics of the 1040 women included in the study are presented in (Table 1). The average age of participants at study entry and mammography was 57.9 years (SD 9.3). Three hundred thirty-five women were premenopausal (mean age 46.8, SD 3.9) and 705 were postmenopausal (mean age 63.1 and SD 5.9), at study entry. The average MD area was 24.9 cm^2^ (SD 22.2) (premenopausal 37.4, SD 24.7; postmenopausal 19.0 SD 18.2), and the average MD change was − 0.8 cm^2^ of dense area per year (SD 3.3) (premenopausal − 1.5, SD 4.3; postmenopausal − 0.5, SD 2.7). On average, 2.8 (median 3.0) mammography screening examinations were available to calculate the annual MD change. The average time spread for the follow-up mammograms were between 12 and 24 months in the study.

**Table 1:** Characteristics of the study population (*n* = 1040) at blood draw and study entry

*Characteristic*	*No.*	*Mean (SD) or %*
Age at blood draw, years	1040	57.9 (9.3)
BMI at study entry, kg/m^2^	1040	25.3 (4.0)
Age at menarche, years	1016	13.2 (1.5)
Ever use of contraceptives, %	1028	83.9
Number of births, *n*	1039	1.9 (1.1)
Age at first birth, years	921	26.8 (5.2)
Postmenopausal, *n*	705	67.8
Age at menopause, years	371	49.6 (5.4)
Alcohol consumption, g/day	1035	6.9 (8.3)
Physical activity, MET-h/d	1040	42.4 (6.4)
Previous use of MHT, %	1040	24.4
Mammographic features		
Mammographic density, dense area, cm^2^	1040	24.9 (22.2)
Mammographic density change, dense area, cm^2^/year	1040	− 0.8 (3.3)
*Circulating hormones*		*Median (SD)*
Progestogens		
Pregnenolone, ng/mL	718	5.3 (10.2)
Progesterone, ng/mL	905	3.9 (22.4)
17OH-progesterone, ng/mL	910	2.1 (11.4)
Androgens		
DHEA, ng/mL	768	22.0 (45.2)
DHEAS, μg/mL	974	1.8 (2.2)
Androstenedione, ng/mL	817	4.8 (24.6)
Testosterone, ng/mL	829	2.0 (11.1)
Free testosterone, pg/mL	824	46.6 (312.0)
Androsterone, ng/mL	711	13.1 (17.0)
Etiocholanolone, ng/mL	654	6.1 (10.4)
Oestrogens		
Oestrone, ng/mL	450	6.3 (21.5)
Oestrone sulphate, ng/mL	938	7.1 (21.3)
Corticoids		
Corticosterone, ng/mL	915	4.1 (14.4)
Aldosterone, ng/mL	565	0.8 (2.5)
11-Deoxycortisol, ng/mL	866	3.2 (15.1)
Cortisol, ng/mL	914	128.8 (61.7)
Cortisone, ng/mL	909	44.7 (35.1)
Peptide hormones and proteins		
Prolactin, ng/mL	1035	15.4 (14.2)
SHBG, μg/mL	1034	4.1 (2.4)

*BMI* body mass index, *DHEA* dehydroepiandrosterone, *DHEAS* dehydroepiandrosterone sulphate, *MET* metabolic equivalent of task, *MHT* menopausal hormone therapy, *SD* standard deviations, *SHBG* sex hormone-binding globulin

We measured 17 hormones from the progestogen (*n* = 3), androgen (*n* = 7), oestrogen (*n* = 2) and corticoid (*n* = 5) pathways, as well as peptide hormones and proteins (*n* = 2) (Table 1). Concentrations of pregnenolone, progesterone, 17OH-progesterone, DHEA, DHEAS, androstenedione, oestrone sulphate and prolactin (all *P* < 0.001); androsterone (*P* = 0.001); and etiocholanolone (*P* = 0.023) were all significantly lower in postmenopausal compared to premenopausal women. The overall range of quantification was between 43.3 and 99.5% (Table 1). Missing values were technical and not associated with menopausal status.

### Hormonal determinants of baseline MD

**Fig. 1:**
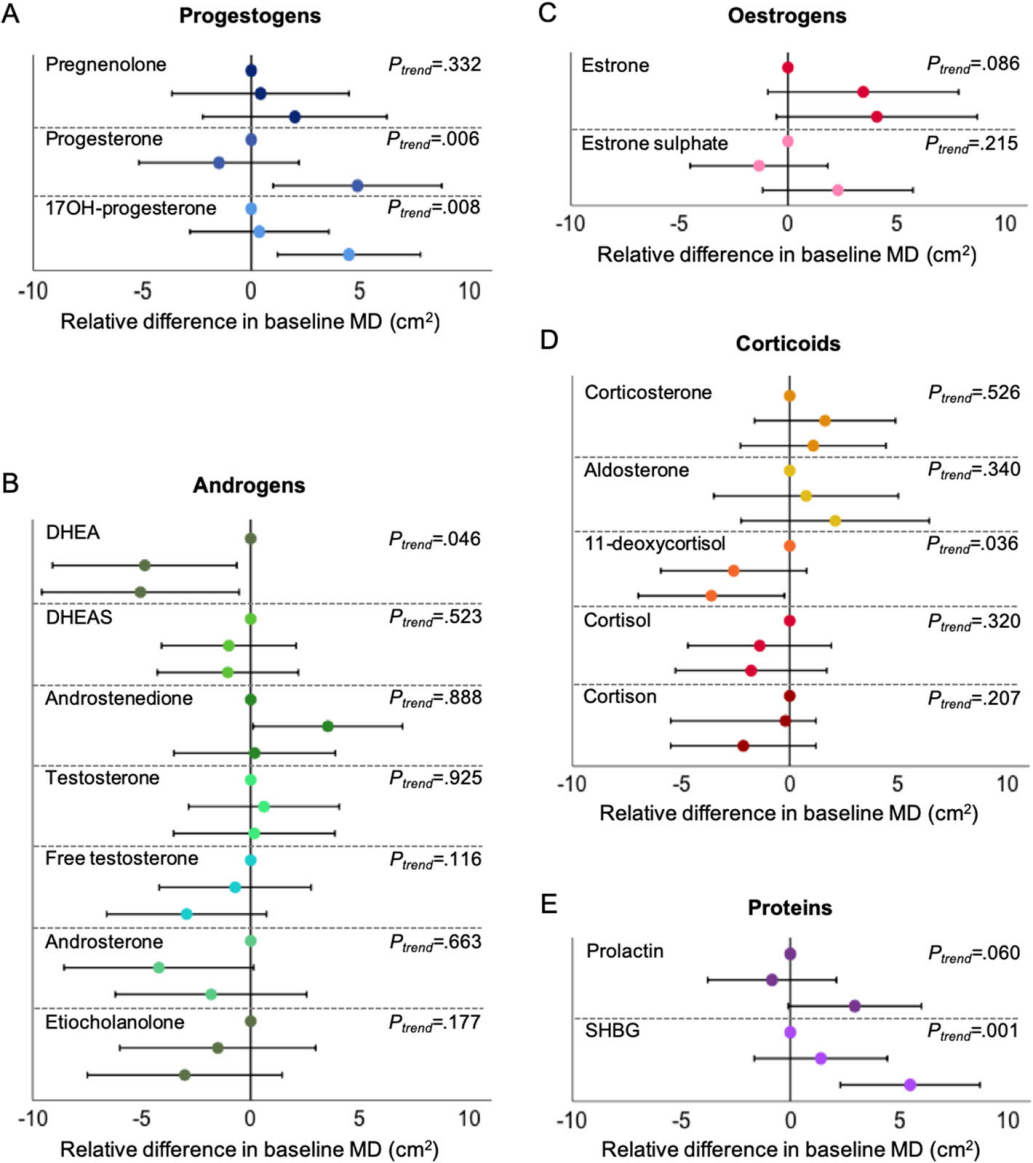
Relative differences in baseline mammographic density (cm^2^) (mean and 95% CI), by tertiles of baseline endogenous plasma hormones at study entry with the lowest tertile (Q1) as reference. **a** Progestogens. **b** Androgens. **c** Oestrogens. **d** Corticoids. **e** Peptide hormones/proteins. Vertical lines represent the median dense area in the entire population (18.5 cm^2^). Models are adjusted for age and BMI at baseline, time of day of blood draw and plasma sample plate number. Two-sided *P* values for the trend of baseline dense area (cm^2^) at blood collection as a dependent continuous variable across tertiles of hormones

The influence of endogenous hormone concentrations on MD (cm^2^) in the entire population is shown in Table 2 and Fig. 1. A doubling of progesterone concentration corresponded to an increase of + 1.29 cm^2^ in baseline MD (*P* < 0.001). Similar was seen for 17OH-progesterone (+ 1.09 cm^2^; *P* = 0.028), oesterone sulphate (+ 1.42 cm^2^; *P* = 0.034), prolactin (+ 2.11 cm^2^; *P* = 0.049) and SHBG (+ 4.18 cm^2^; *P* < 0.001). Women in the highest tertile (Q3) of progesterone had an average baseline MD of 29.26 cm^2^ as compared with 24.39 cm^2^ for women in the lowest tertile (Q1) (*P*_difference_ = 0.014) (Fig. 1; Additional file 1: Table S1). Similar was seen for 17OH-progesterone (Q3: 28.19 cm^2^ versus Q1: 23.71 cm^2^; *P*_difference_ = 0.007) and SHBG (Q3: 28.02 cm^2^ versus Q1: 22.52 cm^2^; *P*_difference_ = 0.001).

**Table 2:** Endogenous hormone determinants of mammographic density area at baseline and area change over time in all 1040 women, not currently using MHT

Determinants	Women, no.	Mammographic dense area		Mammographic dense area change	
		Associations in baseline dense area, cm^2^, *β* estimates (95% CI)*	*P* value^†^	Associations in relative dense area change, cm^2^/year, *β* estimates (95% CI)**	*P* value^††^
**Progestogens**					
Pregnenolone	714	0.48 (− 0.97 to 1.93)	0.514	0.07 (− 0.16 to 0.31)	0.544
Progesterone	898	1.29 (0.57 to 2.01)	< 0.001	− 0.01 (− 0.13 to 0.11)	0.818
17OH-progesterone	903	1.09 (0.12 to 2.07)	0.028	0.07 (− 0.09 to 0.23)	0.368
**Androgens**					
DHEA	764	− 0 .83 (− 2.15 to 0.50)	0.221	0.15 (− 0.07 to 0.37)	0.173
DHEAS	967	− 0.55 (− 2.38 to 1.29)	0.558	− 0.11 (− 0.41 to 0.18)	0.455
Androstenedione	813	− 0.11 (− 1.49 to 1.28)	0.878	0.34 (0.10 to 0.57)	0.005
Testosterone	825	0.11 (− 1.33 to 1.60)	0.877	0.35 (0.11 to 0.59)	0.004
Free testosterone	820	− 0.88 (− 2.14 to 0.38)	0.172	0.31 (0.10 to 0.51)	0.004
Androsterone	708	− 0.12 (− 1.31 to 1.07)	0.849	0.09 (− 0.11 to 0.29)	0.396
Etiocholanolone	651	− 1.15 (− 2.60 to 0.29)	0.118	0.02 (− 0.22 to 0.27)	0.853
**Oestrogens**					
Oestrone	446	0.82 (− 0.35 to 1.98)	0.169	0.07 (− 0.14 to 0.28)	0.535
Oestrone sulphate	931	1.42 (0.10 to 2.73)	0.034	− 0.07 (− 0.28 to 0.15)	0.535
**Corticoids**					
Corticosterone	909	0.05 (− 1.30 to 1.40)	0.944	− 0.09 (− 0.31 to 0.13)	0.425
Aldosterone	562	0.08 (− 1.85 to 2.01)	0.933	0.21 (− 0.10 to 0.53)	0.175
11-Deoxycortisol	860	− 1.33 (− 2.55 to − 0.11)	0.033	0.10 (− 0.10 to 0.30)	0.343
Cortisol	908	− 0.47 (− 3.20 to 2.27)	0.738	0.04 (− 0.41 to 0.48)	0.873
Cortisone	903	− 1.73 (− 3.84 to 0.39)	0.110	0.25 (− 0.09 to 0.60)	0.154
**Peptide hormones**					
Prolactin	1028	2.11 (0.01 to 4.22)	0.049	0.15 (− 0.20 to 0.50)	0.401
SHBG	1028	4.18 (1.91 to 6.45)	< 0.001	− 0.10 (− 0.47 to 0.27)	0.599

*BMI* body mass index, *CI* confidence interval, *DHEA* dehydroepiandrosterone, *DHEAS* dehydroepiandrosterone sulphate, *MET* metabolic equivalent of task, *MHT* menopausal hormone therapy, *SHBG* sex hormone-binding globulin

*Adjusted model: age and BMI at baseline, time of day of blood draw and plasma sample plate number

^†^*P* value is for baseline dense area (cm^2^) at blood collection as a dependent continuous variable by hormones (continuous, natural log-transformed)

**Adjusted model: age, BMI, physical activity (MET-h/d) at baseline, time of day of blood draw and plasma sample plate number

^††^*P* value is for the dense area change (cm^2^/year) as a dependent continuous variable by hormones (continuous, natural log-transformed)

A higher concentration of DHEA and 11-deoxycortisol was inversely associated with baseline MD. Women in Q3 of DHEA had an average baseline MD of 23.59 cm^2^ versus 28.65 cm^2^ in Q1 (*P*_difference_ = 0.029), corresponding to a lower baseline MD of − 1.33 cm^2^ per doubling of hormone (*P* = 0.033). Women in Q3 of 11-deoxycortisol had an average baseline MD of 23.68 cm^2^ versus 27.28 cm^2^ in Q1 (*P*_difference_ = 0.036), corresponding to a lower baseline MD of − 1.33 cm^2^ per doubling of hormone (*P* = 0.033).

When stratifying by menopausal status, progesterone was positively associated with baseline MD among premenopausal (+ 1.78 cm^2^ per doubling of hormone; *P* = 0.004) (Table 3), but not postmenopausal (− 0.07 cm^2^; *P* = 0.888), women (Table 4). SHBG was positively associated with baseline MD in both premenopausal and postmenopausal women (+ 6.58 cm^2^, *P* = 0.004; + 2.60 cm^2^, *P* = 0.042, respectively). Other hormones did not reach statistical significance in the stratified analyses.

**Table 3:** Endogenous hormone determinants of mammographic density area at baseline and area change over time in premenopausal women (*n* = 335), not currently using MHT

Determinants	Women, no.	Mammographic dense area		Mammographic dense area change	
		Associations in baseline dense area, cm^2^, *β* estimates (95% CI)*	*P* value^†^	Associations in relative dense area change, cm^2^/year, *β* estimates (95% CI)**	*P* value^††^
**Progestogens**					
Pregnenolone	230	− 0.16 (− 3.29 to 2.97)	0.919	0.06 (− 0.51 to 0.63)	0.846
Progesterone	299	1.78 (0.58 to 3.00)	0.004	− 0.08 (− 0.31 to 0.14)	0.481
17OH-progesterone	299	1.75 (− 0.15 to 3.66)	0.071	0.17 (− 0.19 to 0.53)	0.362
**Androgens**					
DHEA	255	− 1.33 (− 4.15 to 1.49)	0.353	− 0.10 (− 0.61 to 0.41)	0.689
DHEAS	306	− 3.33 (− 7.49 to 0.82)	0.115	0.06 (− 0.70 to 0.82)	0.872
Androstenedione	270	− 0.45 (− 3.28 to 2.39)	0.758	0.41 (− 0.13 to 0.96)	0.139
Testosterone	267	− 0.81 (− 3.87 to 2.24)	0.602	0.53 (− 0.02 to 1.09)	0.057
Free testosterone	262	− 2.13 (− 4.78 to 0.53)	0.116	0.53 (0.05 to 1.01)	0.030
Androsterone	237	− 0.41 (− 2.92 to 2.10)	0.749	− 0.17 (− 0.63 to 0.29)	0.474
Etiocholanolone	226	− 0.71 (− 3.59 to 2.19)	0.632	− 0.15 (− 0.68 to 0.38)	0.580
**Oestrogens**					
Oestrone	139	0.84 (− 1.79 to 3.47)	0.527	0.16 (− 0.35 to 0.67)	0.537
Oestrone sulphate	301	1.30 (− 1.22 to 3.83)	0.311	− 0.08 (− 0.54 to 0.38)	0.740
**Corticoids**					
Corticosterone	293	0.51 (− 2.14 to 3.15)	0.706	− 0.36 (− 0.85 to 0.12)	0.144
Aldosterone	175	2.05 (− 2.01 to 6.10)	0.321	0.26 (− 0.51 to 1.02)	0.506
11-Deoxycortisol	280	− 1.17 (− 3.60 to 1.27)	0.346	− 0.03 (− 0.48 to 0.42)	0.903
Cortisol	292	− 0.85 (− 6.49 to 4.80)	0.768	− 0.04 (− 1.06 to 0.99)	0.943
Cortisone	290	− 3.35 (− 7.85 to 1.15)	0.144	0.08 (− 0.74 to 0.89)	0.857
**Peptide hormones**					
Prolactin	331	2.35 (− 2.02 to 6.71)	0.291	0.66 (− 0.13 to 1.45)	0.103
SHBG	326	6.58 (2.10 to 11.06)	0.004	− 0.39 (− 1.22 to 0.43)	0.348

*BMI* body mass index, *CI* confidence interval, *DHEA* dehydroepiandrosterone, *DHEAS* dehydroepiandrosterone sulphate, *MET* metabolic equivalent of task, *MHT* menopausal hormone therapy, *SHBG* sex hormone-binding globulin

*Adjusted model: age and BMI at baseline, time of day of blood draw and plasma sample plate number

^†^*P* value is for baseline dense area (cm^2^) at blood collection as a dependent continuous variable by hormones (continuous, natural log-transformed)

**Adjusted model: age, BMI, physical activity (MET-h/d) at baseline, time of day of blood draw and plasma sample plate number

^††^*P* value is for the dense area change (cm^2^/year) as a dependent continuous variable by hormones (continuous, natural log-transformed)

**Table 4:** Endogenous hormone determinants of mammographic density area at baseline and area change over time in postmenopausal women (*n* = 705), not currently using MHT

Determinants	Women, no.	Mammographic dense area		Mammographic dense area change	
		Associations in baseline dense area, cm^2^, *β* estimates (95% CI)*	*P* value^†^	Associations in relative dense area change, cm^2^/year, *β* estimates (95% CI)**	*P* value^††^
**Progestogens**					
Pregnenolone	484	0.53 (− 0.99 to 2.05)	0.493	0.07 (− 0.15 to 0.29)	0.530
Progesterone	599	− 0.07 (− 1.01 to 0.87)	0.888	0.06 (− 0.08 to 0.20)	0.405
17OH-progesterone	604	0.18 (− 0.91 to 1.27)	0.745	0.03 (− 0.13 to 0.19)	0.687
**Androgens**					
DHEA	509	− 0.62 (− 2.00 to 0.77)	0.381	0.35 (0.14 to 0.56)	0.001
DHEAS	661	0.09 (− 1.79 to 1.98)	0.922	− 0.13 (− 0.41 to 0.14)	0.347
Androstenedione	543	− 0.47 (− 1.95 to 1.01)	0.535	0.30 (0.08 to 0.53)	0.008
Testosterone	558	0.51 (− 1.02 to 2.03)	0.513	0.22 (− 0.02 to 0.45)	0.069
Free testosterone	558	− 0.27 (− 1.60 to 1.05)	0.686	0.17 (− 0.03 to 0.37)	0.103
Androsterone	471	− 0.16 (− 1.40 to 1.08)	0.801	0.27 (0.08 to 0.47)	0.006
Etiocholanolone	425	− 1.30 (− 2.84 to 0.23)	0.096	0.20 (− 0.04 to 0.44)	0.103
**Oestrogens**					
Oestrone	307	0.47 (− 0.74 to 1.68)	0.444	0.07 (− 0.14 to 0.28)	0.498
Oestrone sulphate	630	− 0.07 (− 1.59 to 1.46)	0.932	0.02 (− 020 to 0.24)	0.866
**Corticoids**					
Corticosterone	616	− 0.05 (− 1.53 to 1.43)	0.946	0.02 (− 0.20 to 0.24)	0.876
Aldosterone	387	− 0.64 (− 2.70 to 1.42)	0.542	0.22 (− 0.09 to 0.48)	0.182
11-Deoxycortisol	580	− 1.03 (2.37 to 0.29)	0.127	0.12 (− 0.08 to 0.32)	0.226
Cortisol	616	− 0.60 (− 3.54 to 2.34)	0.689	0.08 (− 0.35 to 0.51)	0.717
Cortisone	613	− 1.15 (3.40 to 1.10)	0.315	0.26 (− 0.07 to 0.60)	0.119
**Peptide hormones**					
Prolactin	697	1.05 (− 1.21 to 3.31)	0.361	0.05 (− 0.29 to 0.39)	0.777
SHBG	702	2.60 (0.10 to 5.10)	0.042	0.01 (− 0.36 to 0.39)	0.942

*BMI* body mass index, *CI* confidence interval, *DHEA* dehydroepiandrosterone, *DHEAS* dehydroepiandrosterone sulphate, *MET* metabolic equivalent of task, *MHT* menopausal hormone therapy, *SHBG* sex hormone-binding globulin

*Adjusted model: age and BMI at baseline, time of day of blood draw and plasma sample plate number

^†^*P* value is for baseline dense area (cm^2^) at blood collection as a dependent continuous variable by hormones (continuous, natural log-transformed)

**Adjusted model: age, BMI, physical activity (MET-h/d) at baseline, time of day of blood draw and plasma sample plate number

^††^*P* value is for the dense area change (cm^2^/year) as a dependent continuous variable by hormones (continuous, natural log-transformed)

### Hormonal determinants of MD change

**Fig. 2:**
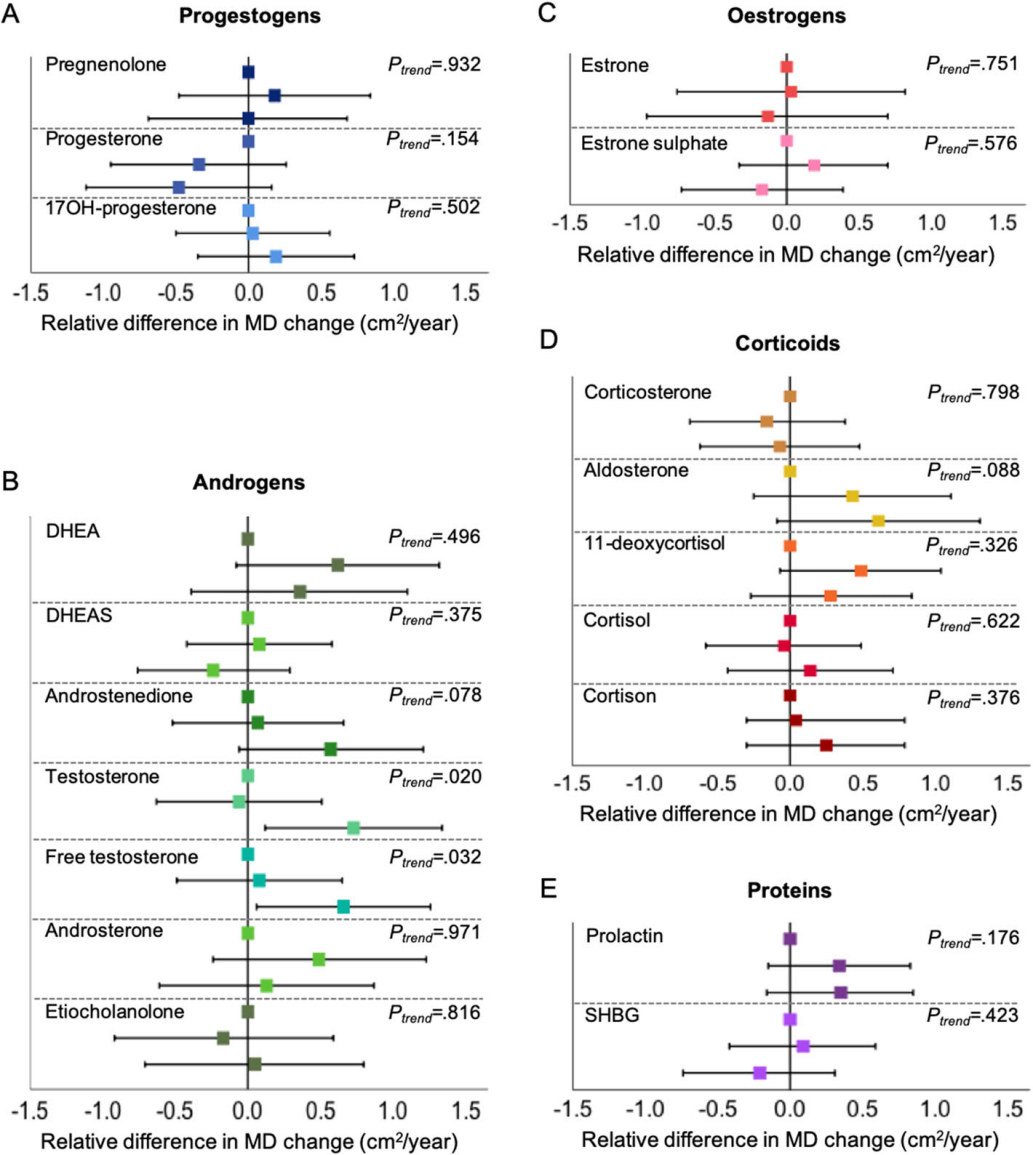
Relative differences in mammographic density change over time (cm^2^/year) (mean and 95% CI), by tertiles of baseline endogenous plasma hormones at study entry with the lowest tertile (Q1) as reference. **a** Progestogens. **b** Androgens. **c** Oestrogens. **d** Corticoids. **e** Peptide hormones/proteins. Vertical lines represent no change in dense area over time. Models are adjusted for age, BMI, physical activity (MET-h/d) at baseline, time of day of blood draw and plasma sample plate number. Two-sided *P* values for trend for dense area change (cm^2^/year) as a dependent continuous variable across tertiles of hormones

The influence of endogenous hormone concentrations on MD change (cm^2^/year) in the entire population is shown in Table 2. Greater concentrations of hormones were associated with MD change for hormones in the androgen pathway. Androstenedione was associated with lesser MD change (0.34 cm^2^/year per doubling of hormone concentration; *P* = 0.005), as was testosterone (0.35 cm^2^/year; *P* = 0.004) and free testosterone (0.31 cm^2^/year; *P* = 0.004) (Table 2). Women in Q3 of testosterone had an average MD change decrease of − 0.38 cm^2^/year versus − 1.11 cm^2^/year for women in Q1 (*P*_difference_ = 0.019) (Fig. 2 and Additional file 1: Table S2). Similarly, women in Q3 of free testosterone had an average MD change of − 0.48 cm^2^/year versus − 1.14 cm^2^/year for women in Q1 (*P*_difference_ = 0.032). Women in Q3 of androstenedione had an average MD change of − 0.48 cm^2^/year versus − 1.05 cm^2^/year women in Q1; however, the difference was not statistically significant (*P*_difference_ = 0.076).

When stratifying by menopausal status, only free testosterone was significantly associated with MD change (0.53 cm^2^/year per doubling of hormone concentration; *P* = 0.030) among premenopausal women (Table 3). Total testosterone was borderline significant associated with MD change (*P* = 0.057). In postmenopausal women, MD change was significantly associated with DHEA (0.34 cm^2^/year; *P* = 0.001), androstenedione (0.30 cm^2^/year; *P* = 0.008) and androsterone (0.27 cm^2^/year; *P* = 0.006) (Table 4). Total testosterone was borderline significant associated with MD change in postmenopausal women (*P* = 0.069).

## Discussion

Using the large, prospective KARMA cohort, we found that several endogenous plasma hormones across the major classes, and proteins, were associated with baseline MD. At the same time, the same markers did not seem to be associated with MD change over time. Our findings suggest that, whereas different hormonal regulators affect baseline MD, MD change however is mainly influenced by the androgens.

We found several hormones to be associated with MD, with the strongest associations between progesterone and MD. Higher levels of progesterone have previously been associated with greater MD [10, 12, 34, 35]. Progestogens play an important part in regulating tissue development and maturation in the young breast, and atrophy and involution of the lobules and ducts during and after menopause [36]. Our data also suggest that sex hormones and MD are not always associated in a linear fashion. Progesterone was associated with and overall increase of + 1.3 cm^2^ in dense area per doubling of concentration by linear associations in multivariable-adjusted models. Women in the top tertile of progesterone had 20% more MD, as compared to those in the lowest tertile. In contrast, oestrone sulphate was somewhat more strongly associated with MD (+ 1.4 cm^2^) by linear models, but women in the top tertile of oestrone sulphate had only 9% more MD than women in the lowest tertile. Our findings, supported by previous studies [10, 37], suggest a more complex association between hormones and breast tissue composition and provide information about the relationship between these risk factors. The non-linear relationship between plasma hormones and MD may also in part explain the lack of association or discrepancy in results between studies.

Stratification by menopausal status suggests clear differences in the association between progesterone and MD; progesterone was strongly associated with overall MD in premenopausal, but not postmenopausal, women, in line with previous findings [10, 12, 34, 35, 38]. Except for oestrone sulphate, hormones from the other pathways did not display the same strong influence by menopausal status. Additionally, adjusting for menstrual cycle did not materially influence the results (data not shown), suggesting that the timing in menstrual cycle does not markedly influence the association between overall MD and endogenous progesterone concentrations.

Most studies [5, 9, 10, 13, 39, 40], including ours, have found SHBG to be positively associated with MD. Here, women in the top tertile of SHBG had 24% higher MD area, as compared to those in the lowest tertile. Our findings support those of Schoemaker and colleagues [13] and suggest that SHBG independently influences MD because the associations remained significant after adjustment for oestrone or testosterone (data not shown). SHBG is a steroid-binding protein and binds both oestrogens and androgens. Its expression is associated with several different diseases (for review see [41]); however, its biological mechanisms remain largely unknown. Meta-analyses suggest that high levels of SHBG are protective against breast cancer [42–44]. The current data suggest that any influence of SHBG on the risk of breast cancer is likely independent of MD. The association between SHBG and MD, biological implications on tumourigenesis and potential clinical implementations need to be further studied.

In contrast to total MD, the MD change over time is not influenced by typical breast cancer risk factors [15, 22, 23]. Factors most strongly associated with MD change are age, BMI and physical activity. We found MD change to be inversely associated with hormones in the androgen pathway only, after adjusting for age, BMI and physical activity. Women in the highest tertile of testosterone had 2.9 times lesser MD change per year compared to those in the lowest tertile of testosterone. Similar results were observed for free testosterone, where those in the highest tertile had 2.4 times lesser annual MD change compared to the lowest tertile. To our knowledge, this is the first study investigating the association between endogenous plasma hormones and MD change.

The androgen pathway, and testosterone in particular, is associated with breast cancer risk [13, 45–48]. One hypothesis could be that the increased risk by higher circulating levels of androgens is mediated through slower MD change over time. Contradicting this hypothesis, we and others have found no statistical evidence for an association between annual MD change and risk of breast cancer [15, 22, 23]. We previously concluded that the risk of breast cancer is dependent on baseline MD, rather than the MD change over time. The interaction between androgens and MD change in relation to breast cancer risk was not the scoop of the study and need to be further studied. Nonetheless, our findings suggest that although endogenous androgens influence the rate of annual MD change, there are likely additional mechanisms driving the risk of breast cancer associated with testosterone.

We and others have previously shown that sex hormones and average MD are independent risk factors for breast cancer [13, 48, 49]. Women in the highest tertile of both sex hormone levels and MD were at 2.4- to 7.8-fold greater risk of breast cancer, compared to those in the lowest tertile. Accordingly, hormones may act both as independent risk factors, but they may also influence breast tissue composition. Hypothetically, the same may be true for the associations between androgens, MD change and breast cancer risk. This emphasises the complexity of risk factors and their mechanisms of action and warrants more attention.

This study has some limitations. Although the KARMA cohort is comprehensive and that this study is among the largest to evaluate the associations between endogenous hormones and mammographic density, hormone data was missing for some participants due to technical error. The missing data likely decrease the power of the analyses and may dilute the associations, in particular, for stratified analyses comparing premenopausal and postmenopausal women. Furthermore, some steroids from the different pathways were not included in our method of analysis or were missing to a larger extent, thus reducing the possibility to generalise the findings between the pathways. Furthermore, we only had baseline plasma hormone concentrations and questionnaire data to which we compare the follow-up mammograms. Follow-up plasma hormone concentrations, updated menopausal status and updated information on MHT use may have enabled further perspectives. Finally, all exposure data is self-reported, which may result in measurement bias. However, exposure data, mammograms and blood samples were collected at the same time at KARMA study entry and it is not likely that the participants knew about their mammographic density or future density change at the time of answering the questionnaire. Furthermore, a non-differential misclassification of exposures would dilute, not strengthen, the reported associations.

The strengths of our study are the large number of samples and the fast, sensitive and reliable UPSFC-MS/MS method for simultaneous quantification of 17 endogenous steroids [30]. Some hormones display a circadian rhythm; we thus included the time of day of blood draw in our models. Furthermore, the KARMA study provides centralised collection and handling of mammograms and blood samples, the quantitative assessment of mammographic density and density change by STRATUS, and collection of background information of all participants [31]. For example, it has been abundantly shown in the literature that MHT influences the total MD; the comprehensive KARMA questionnaire data enabled easy selection and exclusion of participants with current MHT at time of blood draw and mammogram.

## Conclusion

In this large prospective cohort study, endogenous hormones from the progesterone, oestrogen and corticoid pathways, as well as prolactin and SHBG, were all associated with baseline MD. The same hormones were however not associated with MD change over time. In contrast, MD change was associated with hormones from the androgen pathway. Higher plasma concentrations of androgens, and testosterone in particular, were associated with slower MD change over time. Our findings suggest that, whereas different hormonal regulators drive baseline MD, MD change is mainly affected by the androgens. This study emphasises the complexity of risk factors and their mechanisms of action. The association between endogenous hormones, MD and MD change, need to be replicated in independent studies. Nonetheless, the potential use and clinical implementations for hormones as determinants of MD and MD change warrant more attention.

## Supplementary information

**Additional file 1.** Additional Results. (Tables S1-S2). **Table S1.** Endogenous hormone determinants of mammographic density area at baseline in all 1040 women, not currently using MHT. **Table S2.** Endogenous hormone determinants of mammographic density area change per year in all 1040 women, not currently using MHT.

## Data Availability

The datasets used and/or analysed during the current study are available from the corresponding author on reasonable request.
