# Supplementary material for: Hormonal determinants of mammographic density and density change
Source: Breast Cancer Res. 2020 Aug 26;22:95. doi: 10.1186/s13058-020-01332-4 (PMC7449090; doi:10.1186/s13058-020-01332-4)
Supplement: Supplementary file 1 — Additional file 1. Additional Results. (Tables S1-S2). Table S1. Endogenous hormone determinants of mammographic density area at baseline in all 1040 women, not currently using MHT. Table S2. Endogenous hormone determinants of mammographic density area change per year in all 1040 women, not currently using MHT. [file 13058_2020_1332_MOESM1_ESM.docx]

**Additional Table S1.** Endogenous hormone determinants of mammographic density area at baseline in all 1,040 women, not currently using MHT.

| Determinants |  | | Women  No. (%) | | Mean dense area at baseline in cm^2^  (95% CI)^†^ | | Relative difference in mean dense area in cm^2^ (95% CI)^†^ | | *P*-value^†^ | | *P*-trend^††^ | |
| --- | --- | --- | --- | --- | --- | --- | --- | --- | --- | --- | --- | --- |
| **Progestogens** |  | |  | |  | |  | |  | |  | |
| Pregnenolone | Q1 | | 239 | | 24.02 (21.13 to 26.91) | | Ref. | |  | |  | |
|  | Q2 | | 239 | | 24.44 (21.81 to 27.07) | | 0.42 (-3.64 to 4.48) | | 0.838 | |  | |
|  | Q3 | | 236 | | 26.02 (23.29 to 28.74) | | 2.00 (-2.23 to 6.22) | | 0.354 | |  | |
|  | Trend | | 714 | |  | |  | |  | | 0.332 | |
| Progesterone | Q1 | | 298 | | 24.39 (21.77 to 27.01) | | Ref. | |  | |  | |
|  | Q2 | | 303 | | 22.90 (20.56 to 25.24) | | -1.49 (-5.16 to 2.19) | | 0.427 | |  | |
|  | Q3 | | 297 | | 29.26 (26.80 to 31.72) | | 4.87 (-1.20 to 8.74) | | 0.014 | |  | |
|  | Trend | | 898 | |  | |  | |  | | 0.006 | |
| 17OH-progesterone | Q1 | | 300 | | 23.71 (21.44 to 25.98) | | Ref. | |  | |  | |
|  | Q2 | | 304 | | 24.07 (21.82 to 26.33) | | 0.37 (-2.82 to 3.55) | | 0.822 | |  | |
|  | Q3 | | 299 | | 28.19 (25.87 to 30.50) | | 4.48 (1.20 to 7.76) | | 0.007 | |  | |
|  | Trend | | 903 | |  | |  | |  | | 0.008 | |
| **Androgens** |  | |  | |  | |  | |  | |  | |
| DHEA | Q1 | | 255 | | 28.65 (25.63 to 31.67) | | Ref. | |  | |  | |
|  | Q2 | | 255 | | 23.79 (21.20 to 26.38) | | -4.86 (-9.09 to -0.64) | | 0.024 | |  | |
|  | Q3 | | 254 | | 23.59 (20.83 to 26.35) | | -5.06 (-9.58 to -0.53) | | 0.029 | |  | |
|  | Trend | | 764 | |  | |  | |  | | 0.046 | |
| DHEAS | Q1 | | 323 | | 25.50 (23.29 to 27.71) | | Ref. | |  | |  | |
|  | Q2 | | 320 | | 24.50 (22.34 to 26.66) | | -1.00 (-4.09 to 2.08) | | 0.524 | |  | |
|  | Q3 | | 324 | | 24.45 (22.23 to 26.67) | | -1.05 (-4.28 to 2.19) | | 0.525 | |  | |
|  | Trend | | 967 | |  | |  | |  | | 0.523 | |
| Androstenedione | Q1 | | 271 | | 23.92 (21.44 to 26.41) | | Ref. | |  | |  | |
|  | Q2 | | 273 | | 27.46 (25.10 to 29.83) | | 3.54 (0.11 to 6.97) | | 0.043 | |  | |
|  | Q3 | | 269 | | 24.11 (21.59 to 26.62) | | 0.19 (-3.52 to 3.89) | | 0.922 | |  | |
|  | Trend | | 813 | |  | |  | |  | | 0.888 | |
| Testosterone | Q1 | | 277 | | 25.07 (22.58 to 27.56) | | Ref. | |  | |  | |
|  | Q2 | | 276 | | 25.67 (23.28 to 28.07) | | 0.61 (-2.85 to 4.06) | | 0.730 | |  | |
|  | Q3 | | 272 | | 25.24 (22.71 to 27.77) | | 0.17 (-3.54 to 3.87) | | 0.929 | |  | |
|  | Trend | | 825 | |  | |  | |  | | 0.925 | |
| Free testosterone | Q1 | | 274 | | 26.44 (23.95 to 28.94) | | Ref. | |  | |  | |
|  | Q2 | | 275 | | 25.73 (23.32 to 28.14) | | -0.71 (-4.19 to 2.77) | | 0.687 | |  | |
|  | Q3 | | 271 | | 23.51 (21.00 to 26.02) | | -2.94 (-6.60 to 0.73) | | 0.116 | |  | |
|  | Trend | | 820 | |  | |  | |  | | 0.116 | |
| Androsterone | Q1 | | 237 | | 27.28 (24.25 to 30.30) | | Ref. | |  | |  | |
|  | Q2 | | 237 | | 23.07 (20.40 to 25.74) | | -4.21 (-8.56 to 0.14) | | 0.058 | |  | |
|  | Q3 | | 234 | | 25.46 (22.76 to 28.16) | | -1.82 (-6.21 to 2.57) | | 0.417 | |  | |
|  | Trend | | 708 | |  | |  | |  | | 0.663 | |
| Etiocholanolone | Q1 | | 218 | | 27.45 (24.33 to 30.56) | | Ref. | |  | |  | |
|  | Q2 | | 217 | | 25.94 (23.11 to 28.77) | | -1.51 (-6.00 to 2.99) | | 0.510 | |  | |
|  | Q3 | | 216 | | 24.42 (21.61 to 27.24) | | -3.02 (-7.49 to 1.44) | | 0.184 | |  | |
|  | Trend | | 651 | |  | |  | |  | | 0.177 | |
| **Oestrogens** |  | |  | |  | |  | |  | |  | |
| Oestrone | Q1 | | 148 | | 19.95 (16.79 to 23.11) | | Ref. | |  | |  | |
|  | Q2 | | 149 | | 23.42 (20.42 to 26.41) | | 3.47 (-0.93 to 7.86) | | 0.122 | |  | |
|  | Q3 | | 149 | | 24.04 (20.93 to 27.15) | | 4.09 (-0.54 to 8.72) | | 0.083 | |  | |
|  | Trend | | 446 | |  | |  | |  | | 0.086 | |
|  |  | |  | |  | |  | | *Table cont.* | | | |
| *Table cont.* |  |  | |  | |  | |  | |  | |  |
| Oestrone sulphate | Q1 | | 310 | | 24.72 (22.42 to 27.03) | | Ref. | |  | |  | |
|  | Q2 | | 311 | | 23.39 (21.18 to 25.60) | | -1.34 (-4.51 to 1.84) | | 0.409 | |  | |
|  | Q3 | | 310 | | 27.02 (24.67 to 29.37) | | 2.30 (-1.16 to 5.76) | | 0.193 | |  | |
|  | Trend | | 931 | |  | |  | |  | | 0.215 | |
| **Corticoids** |  | |  | |  | |  | |  | |  | |
| Corticosterone | Q1 | | 302 | | 24.14 (21.81 to 26.46) | | Ref. | |  | |  | |
|  | Q2 | | 304 | | 25.77 (23.50 to 28.03) | | 1.63 (-1.62 to 4.88) | | 0.325 | |  | |
|  | Q3 | | 303 | | 25.22 (22.91 to 27.54) | | 1.09 (-2.26 to 4.44) | | 0.524 | |  | |
|  | Trend | | 909 | |  | |  | |  | | 0.526 | |
| Aldosterone | Q1 | | 186 | | 23.92 (20.90 to 26.95) | | Ref. | |  | |  | |
|  | Q2 | | 188 | | 24.68 (21.75 to 27.61) | | 0.76 (-3.49 to 5.00) | | 0.727 | |  | |
|  | Q3 | | 188 | | 26.02 (23.04 to 29.00) | | 2.10 (-2.24 to 6.43) | | 0.342 | |  | |
|  | Trend | | 562 | |  | |  | |  | | 0.340 | |
| 11-deoxycortisol | Q1 | | 286 | | 27.28 (24.91 to 29.66) | | Ref. | |  | |  | |
|  | Q2 | | 289 | | 24.71 (22.36 to 27.05) | | -2.58 (-5.94 to 0.78) | | 0.133 | |  | |
|  | Q3 | | 285 | | 23.68 (21.31 to 26.04) | | -3.61 (-6.97 to -0.24) | | 0.036 | |  | |
|  | Trend | | 860 | |  | |  | |  | | 0.036 | |
| Cortisol | Q1 | | 300 | | 26.04 (23.66 to 28.42) | | Ref. | |  | |  | |
|  | Q2 | | 305 | | 24.66 (22.39 to 26.92) | | -1.38 (-4.68 to 1.92) | | 0.412 | |  | |
|  | Q3 | | 303 | | 24.26 (21.90 to 26.62) | | -1.77 (-5.26 to 1.71) | | 0.318 | |  | |
|  | Trend | | 908 | |  | |  | |  | | 0.320 | |
| Cortisone | Q1 | | 299 | | 25.80 (23.46 to 28.13) | | Ref. | |  | |  | |
|  | Q2 | | 302 | | 25.60 (23.33 to 27.88) | | -0.19 (-5.48 to 1.21) | | 0.908 | |  | |
|  | Q3 | | 302 | | 23.66 (21.35 to 25.97) | | -2.14 (-5.48 to 1.21) | | 0.211 | |  | |
|  | Trend | | 903 | |  | |  | |  | | 0.207 | |
| **Peptide hormones** |  | |  | |  | |  | |  | |  | |
| Prolactin | Q1 | | 344 | | 24.17 (22.06 to 26.28) | | Ref. | |  | |  | |
|  | Q2 | | 343 | | 23.33 (21.26 to 25.41) | | -0.84 (-3.80 to 2.12) | | 0.578 | |  | |
|  | Q3 | | 341 | | 27.13 (25.00 to 29.26) | | 2.96 (-0.10 to 6.02) | | 0.058 | |  | |
|  | Trend | | 1028 | |  | |  | |  | | 0.060 | |
| SHBG | Q1 | | 344 | | 22.52 (20.33 to 24.70) | | Ref. | |  | |  | |
|  | Q2 | | 342 | | 23.92 (21.82 to 26.02) | | 1.40 (-1.65 to 4.45) | | 0.367 | |  | |
|  | Q3 | | 342 | | 28.02 (25.84 to 30.19) | | 5.50 (2.30 to 8.70) | | 0.001 | |  | |
|  | Trend | | 1028 | |  | |  | |  | | 0.001 | |

Abbreviations: BMI, body mass index; CI, confidence interval; DHEA, dehydroepiandrosterone; DHEAS, dehydroepiandrosterone sulphate; MHT, menopausal hormone therapy; Ref., reference; SHBG, sex hormone binding globulin.

* Adjusted model: age and BMI at baseline, time of day of blood draw and plasma sample plate number.

^†^ *P*-value is for the relative difference in mean baseline dense area (cm^2^) compared to the reference group; tests were performed at the two-sided  0.05 significance level.

^††^ *P*-value of linear trend for dense area (cm^2^) as dependent continuous variable across quartiles of hormones; tests were performed at the two-sided  0.05 significance level.

**Additional Table S2.** Endogenous hormone determinants of mammographic density area change per year in all 1,040 women, not currently using MHT.

| Determinants |  | Women  No. (%) | Mean dense area change in cm^2^/y  (95% CI)* | Relative change of dense area in cm^2^/y (95% CI)* | *P*-value^†^ | *P*-trend^††^ |
| --- | --- | --- | --- | --- | --- | --- |
| **Progestogens** |  |  |  |  |  |  |
| Pregnenolone | Q1 | 179 | -1.01 (-1.47 to -0.54) | Ref. |  |  |
|  | Q2 | 179 | -0.82 (-1.25 to -0.40) | 0.18 (-0.48 to 0.84) | 0.587 |  |
|  | Q3 | 180 | -1.01 (-1.45 to -0.57) | 0.00 (-0.69 to 0.68) | 0.991 |  |
|  | Trend | 714 |  |  |  | 0.932 |
| Progesterone | Q1 | 227 | -0.59 (-1.02 to -0.16) | Ref. |  |  |
|  | Q2 | 223 | -0.93 (-1.32 to -0.54) | -0.34 (-0.95 to 0.26) | 0.268 |  |
|  | Q3 | 226 | -1.06 (-1.47 to -0.66) | -0.48 (-1.12 to 0.16) | 0.144 |  |
|  | Trend | 898 |  |  |  | 0.154 |
| 17OH-progesterone | Q1 | 227 | -0.93 (-1.31 to -0.56) | Ref. |  |  |
|  | Q2 | 227 | -0.91 (-1.28 to -0.53) | 0.03 (-0.50 to 0.56) | 0.918 |  |
|  | Q3 | 226 | -0.75 (-1.13 to -0.36) | 0.19 (-0.35 to 0.73) | 0.497 |  |
|  | Trend | 903 |  |  |  | 0.502 |
| **Androgens** |  |  |  |  |  |  |
| DHEA | Q1 | 191 | -1.13 (-1.63 to -0.63) | Ref. |  |  |
|  | Q2 | 191 | -0.51 (-0.94 to -0.08) | 0.62 (-0.08 to 1.32) | 0.081 |  |
|  | Q3 | 190 | -0.78 (-1.23 to -0.32) | 0.36 (-0.39 to 1.10) | 0.352 |  |
|  | Trend | 764 |  |  |  | 0.496 |
| DHEAS | Q1 | 242 | -0.73 (-1.09 to -0.37) | Ref. |  |  |
|  | Q2 | 241 | -0.65 (-1.00 to -0.30) | 0.08 (-0.42 to 0.58) | 0.759 |  |
|  | Q3 | 241 | -0.97 (-1.33 to -0.61) | -0.24 (-0.76 to 0.29) | 0.371 |  |
|  | Trend | 967 |  |  |  | 0.375 |
| Androstenedione | Q1 | 203 | -1.05 (-1.47 to -0.62) | Ref. |  |  |
|  | Q2 | 204 | -0.98 (-1.38 to -0.57) | 0.07 (-0.52 to 0.66) | 0.814 |  |
|  | Q3 | 204 | -0.48 (-0.90 to -0.05) | 0.57 (-0.06 to 1.21) | 0.076 |  |
|  | Trend | 813 |  |  |  | 0.078 |
| Testosterone | Q1 | 207 | -1.11 (-1.52 to -0.70) | Ref. |  |  |
|  | Q2 | 207 | -1.17 (-1.56 to -0.77) | -0.06 (-0.63 to 0.51) | 0.837 |  |
|  | Q3 | 207 | -0.38 (-0.79 to 0.04) | 0.73 (0.12 to 1.34) | 0.019 |  |
|  | Trend | 825 |  |  |  | 0.020 |
| Free testosterone | Q1 | 206 | -1.14 (-1.55 to -0.73) | Ref. |  |  |
|  | Q2 | 206 | -1.06 (-1.46 to -0.67) | 0.08 (-0.49 to 0.65) | 0.785 |  |
|  | Q3 | 205 | -0.48 (-0.90 to -0.07) | 0.66 (0.06 to 1.26) | 0.032 |  |
|  | Trend | 820 |  |  |  | 0.032 |
| Androsterone | Q1 | 177 | -1.03 (-1.54 to -0.52) | Ref. |  |  |
|  | Q2 | 178 | -0.53 (-0.98 to -0.08) | 0.49 (-0.24 to 1.23) | 0.186 |  |
|  | Q3 | 177 | -0.90 (-1.36 to -0.45) | 0.13 (-0.61 to 0.87) | 0.739 |  |
|  | Trend | 708 |  |  |  | 0.971 |
| Etiocholanolone | Q1 | 163 | -0.79 (-1.32 to -0.27) | Ref. |  |  |
|  | Q2 | 164 | -0.96 (-1.43 to -0.48) | -0.17 (-0.92 to 0.59) | 0.669 |  |
|  | Q3 | 162 | -0.75 (-1.22 to -0.27) | 0.05 (-0.71 to 0.80) | 0.904 |  |
|  | Trend | 651 |  |  |  | 0.816 |
| **Oestrogens** |  |  |  |  |  |  |
| Oestrone | Q1 | 110 | -0.50 (-1.07 to 0.07) | Ref. |  |  |
|  | Q2 | 112 | -0.48 (-1.02 to 0.06) | 0.03 (-0.76 to 0.82) | 0.944 |  |
|  | Q3 | 113 | -0.64 (-1.20 to -0.08) | -0.13 (-0.97 to 0.70) | 0.756 |  |
|  | Trend | 446 |  |  |  | 0.751 |
|  |  |  |  |  | *Table cont.* | |
| *Table cont.* |  |  |  |  |  |  |
| Oestrone sulphate | Q1 | 231 | -0.82 (-1.20 to -0.45) | Ref. |  |  |
|  | Q2 | 235 | -0.63 (-0.99 to -0.28) | 0.19 (-0.33 to 0.70) | 0.471 |  |
|  | Q3 | 234 | -0.99 (-1.38 to -0.61) | -0.17 (-0.73 to 0.39) | 0.548 |  |
|  | Trend | 931 |  |  |  | 0.576 |
| **Corticoids** |  |  |  |  |  |  |
| Corticosterone | Q1 | 226 | -0.75 (-1.13 to -0.37) | Ref. |  |  |
|  | Q2 | 228 | -0.91 (-1.28 to -0.54) | -0.16 (-0.69 to 0.38) | 0.568 |  |
|  | Q3 | 228 | -0.83 (-1.20 to -0.45) | -0.07 (-0.62 to 0.48) | 0.795 |  |
|  | Trend | 909 |  |  |  | 0.798 |
| Aldosterone | Q1 | 139 | -0.98 (-1.47 to -0.50) | Ref. |  |  |
|  | Q2 | 140 | -0.55 (-1.03 to -0.08) | 0.43 (-0.25 to 1.11) | 0.219 |  |
|  | Q3 | 142 | -0.37 (-0.85 to 0.11) | 0.61 (-0.09 to 1.31) | 0.087 |  |
|  | Trend | 562 |  |  |  | 0.088 |
| 11-deoxycortisol | Q1 | 214 | -1.09 (-1.48 to -0.70) | Ref. |  |  |
|  | Q2 | 217 | -0.60 (-0.99 to -0.22) | 0.49 (-0.07 to 1.04) | 0.085 |  |
|  | Q3 | 216 | -0.81 (-1.20 to -0.42) | 0.28 (-0.27 to 0.84) | 0.317 |  |
|  | Trend | 860 |  |  |  | 0.326 |
| Cortisol | Q1 | 225 | -0.85 (-1.23 to -0.46) | Ref. |  |  |
|  | Q2 | 228 | -0.89 (-1.26 to -0.52) | -0.04 (-0.58 to 0.49) | 0.872 |  |
|  | Q3 | 229 | -0.71 (-1.09 to -0.32) | 0.14 (-0.43 to 0.71) | 0.626 |  |
|  | Trend | 908 |  |  |  | 0.622 |
| Cortisone | Q1 | 223 | -0.90 (-1.28 to -0.52) | Ref. |  |  |
|  | Q2 | 226 | -0.86 (-1.23 to -0.49) | 0.04 (-0.30 to 0.79) | 0.879 |  |
|  | Q3 | 228 | -0.65 (-1.03 to -0.28) | 0.25 (-0.30 to 0.79) | 0.371 |  |
|  | Trend | 903 |  |  |  | 0.367 |
| **Peptide hormones** |  |  |  |  |  |  |
| Prolactin | Q1 | 257 | -1.04 (-1.39 to -0.69) | Ref. |  |  |
|  | Q2 | 259 | -0.70 (-1.04 to -0.36) | 0.34 (-0.15 to 0.83) | 0.171 |  |
|  | Q3 | 258 | -0.69 (-1.05 to -0.34) | 0.35 (-0.16 to 0.85) | 0.179 |  |
|  | Trend | 1028 |  |  |  | 0.176 |
| SHBG | Q1 | 258 | -0.79 (-1.15 to -0.43) | Ref. |  |  |
|  | Q2 | 257 | -0.70 (-1.05 to -0.36) | 0.09 (-0.42 to 0.59) | 0.737 |  |
|  | Q3 | 257 | -1.00 (-1.36 to -0.64) | -0.21 (-0.74 to 0.31) | 0.429 |  |
|  | Trend | 1028 |  |  |  |  |

Abbreviations: BMI, body mass index; CI, confidence interval; DHEA, dehydroepiandrosterone; DHEAS, dehydroepiandrosterone sulphate; MET, metabolic equivalent of task; MHT, menopausal hormone therapy; Ref., reference; SHBG, sex hormone binding globulin.

* Adjusted model: age, BMI, and physical activity (MET-h/d) at baseline, time of day of blood draw and plasma sample plate number.

^†^ *P*-value is for the relative dense area change in cm^2^/y compared to the reference group; tests were performed at the two-sided  0.05 significance level.

^††^ *P*- value of linear trend for dense area change (cm^2^/year) as dependent continuous variable across quartiles of hormones; tests were performed at the two-sided  0.05 significance level.
